# Terlipressin in paediatric hepatorenal syndrome-acute kidney injury (HRS-AKI)

**DOI:** 10.1007/s00467-026-07205-w

**Published:** 2026-02-19

**Authors:** Emma C. Alexander, Benjamin Wyness, Kevin Moore, Francisco Flores, Akash Deep

**Affiliations:** 1 Paediatric Intensive Care Unit, King’s College Hospital NHS Foundation Trust, Denmark Hill, London, UK; 2 Paediatric Critical Care Research Group, Imperial College London, London, UK; 3 UCL Institute for Liver and Digestive Health, Royal Free Campus, London, UK; 4 Division of Nephrology and Hypertension, Cincinnati Children’s Hospital, Cincinnati, OH USA; 5 Department of Women and Children’s Health, School of Life Course Sciences, King’s College London, London, UK

**Keywords:** Hepatorenal syndrome, Acute kidney injury, Chronic liver disease, Terlipressin, Vasopressors

## Abstract

Children with chronic liver disease are at increased risk of acute kidney injury (AKI), which could be non-hepatorenal syndrome AKI (non-HRS-AKI) or hepatorenal syndrome AKI (HRS-AKI). Approximately 5–10% of these children develop HRS-AKI. In this cohort, portal hypertension leads to splanchnic vasodilatation, reduced mean arterial pressure, and compensatory renal arteriolar vasoconstriction. The reduction of mean arterial pressure and renal vasoconstriction reduces renal perfusion, causing functional renal failure and AKI (acute kidney injury). HRS-AKI may be precipitated by sepsis or inflammation, which may impact cardiac function in patients with cirrhotic cardiomyopathy. While liver transplantation remains curative, adult randomised controlled trials have demonstrated that treatment with terlipressin can potentially reverse HRS-AKI. Limited paediatric studies also suggest terlipressin may also be effective in children, though effects on mortality remain unexplored. In this review, we explore the prevalence and pathophysiology of HRS-AKI in paediatric liver disease, summarise evidence from adult trials supporting terlipressin use, highlight cautions and contraindications associated with its use in adults and children, and propose principles for its safe prescription. Additionally, we outline future research priorities, including the potential role of renal biomarkers in managing this high-risk population.

**Graphical Abstract:**

**Figure:**
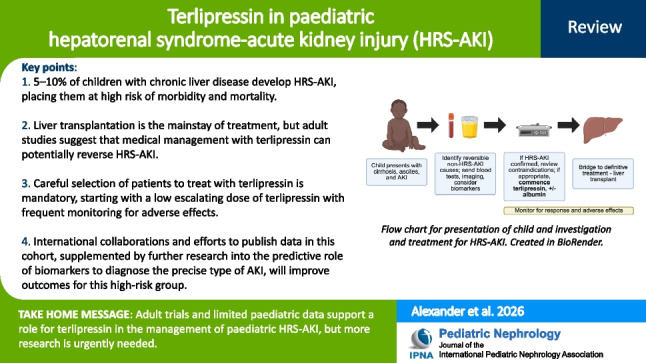
A higher resolution version of the Graphical abstract is available as Supplementary information.

**Supplementary Information:**

The online version contains supplementary material available at 10.1007/s00467-026-07205-w.

## Background

Children with chronic liver disease are at an increased risk of acute kidney injury (AKI) [1]. Causes of AKI in these patients include hypovolaemia (due to gastrointestinal (GI) bleeds, GI losses, overuse of diuretics), sepsis, and intrinsic renal causes, such as acute tubular necrosis, glomerulonephritis, and nephrotoxic drugs. These causes are termed non-hepatorenal syndrome (non-HRS)-AKI. In a study of 84 children with acute-on-chronic liver failure (ACLF), 19 (22.6%) developed AKI [2]. AKI was associated with poor outcomes; of children who developed AKI, 10 (52.6%) died, and only 5 (26.3%) survived with native liver [2]. This makes AKI in these children especially important to recognise and manage.

Hepatorenal syndrome (HRS) is a specific form of renal dysfunction which occurs in patients with liver disease, particularly in those with cirrhosis and ascites. It occurs due to a significant reduction in kidney perfusion secondary to hepatic dysfunction [3]. It is estimated that HRS-AKI occurs in 5% of children with chronic liver disease prior to liver transplantation [3]. HRS-AKI used to be a diagnosis of exclusion amongst patients with chronic liver disease presenting with AKI. However, according to the recent definition as established in the 2023 Acute Disease Quality Initiative (ADQI) and International Club of Ascites (ICA) joint multidisciplinary consensus meeting (**Box 1**), HRS-AKI can co-exist with other pathologies. This meeting had representation from nephrology, critical care, hepatology, transplant surgery, paediatrics, and pharmacology [4]. Though this meeting primarily focused on adult patients, the consensus definition acknowledges that the main pathophysiologic event which triggers the cascade of hemodynamic and inflammatory changes in these patients is portal hypertension, secondary to cirrhosis. Though aetiologies leading to cirrhosis may differ between adults and children, once cirrhosis is established, the downstream effects of portal hypertension are the same, and management principles are therefore similar.

**Table:** 

Box 1: Definition of HRS-AKI from the 2023 Acute Disease Quality Initiative (ADQI) and International Club of Ascites (ICA) joint multidisciplinary consensus meeting
• Cirrhosis with ascites • Increase in serum creatinine ≥ 0.3 mg/dl (26.5 umol/L) within 48 h or ≥ 50% from baseline value known or presumed to have occurred within the prior 7 days and/or urinary output ≤ 0.5 ml/kg/hour for ≥ 6 h • Absence of improvement in serum creatinine and/or urine output within 24 h following adequate volume resuscitation (when clinically indicated) • Absence of strong evidence for an alternative explanation as the primary cause of AKI (e.g. septic shock requiring vasopressors, drug-induced AKI, obstruction, or acute glomerular injury)

HRS-AKI should be distinguished from non-HRS-AKI, namely a type of kidney dysfunction that occurs in people with advanced liver disease but does not meet the criteria for HRS-AKI, though this entity was not formally recognised in the ADQI/ICA consensus meeting. ‘Non-HRS AKI’ clinically is used to mean AKI in a cirrhotic patient that is *not* due to HRS (e.g., sepsis, ATN, dehydration, drug-induced).

ADQI/ICA 2023 replaced the former HRS Type 1 and Type 2 terminology with a time-course based system: HRS-AKI, HRS-AKD (acute kidney disease), and HRS-CKD (chronic kidney disease). HRS-AKD and HRS-CKD reflect renal disease of less acute onset/a more prolonged duration, a formerly termed HRS Type 2 [5–7].

It is specifically important to recognise the aetiology of AKI, especially HRS-AKI, because HRS-AKI is associated with worse outcomes (which have been well-characterised in adult studies) and has different management strategies. However, paediatric-specific data are lacking, which is important, since the aetiology of chronic liver disease in paediatrics (most commonly secondary to biliary atresia) does not match adult cohorts, where alcoholic liver disease and metabolic dysfunction-associated steatotic liver disease (MASLD) predominate [8]. In addition, compared to pre-renal causes of AKI which respond well to treatment with fluids, HRS-AKI does not respond to treatment with fluids alone.

In this review, we will discuss the evidence for HRS in paediatric patients, whilst extrapolating useful data from adult studies where required. We will then review the role of the vasoconstrictor, terlipressin, in the management of paediatric HRS.

## Pathophysiology of HRS-AKI

Various factors contribute to the development of HRS-AKI. Cirrhosis leads to portal hypertension and subsequent splanchnic arterial vasodilatation. Splanchnic vasodilatation is driven by the release of vasodilators and inflammatory cytokines in the context of bacterial translocation, secondary to increased portal pressure [9, 10]. Potential vasodilatory mediators include nitric oxide [11], glucagon [12], and prostacyclin [13]. The release of these mediators results in reduced effective arterial blood volume and mean arterial pressure, triggering compensatory activation of the renin–angiotensin–aldosterone system (RAAS) and the sympathetic nervous system [3, 14] to maintain blood pressure. This compensatory activation leads to sodium and water retention, contributing to ascites. However, the sustained activation of the RAAS and sympathetic nervous system leads to profound renal vasoconstriction, which is insufficiently counterbalanced by intrarenal vasodilatory mediators including prostaglandins. This means that in practice, for a given blood pressure in patients with HRS, there is reduced renal blood flow and a reduced glomerular filtration rate [5]. This renal hypoperfusion results in the functional renal injury that is characteristic of HRS-AKI [6].

There is increasing evidence that systemic inflammation plays an important role in HRS-AKI. In a study of 161 hospitalised adults with decompensated cirrhosis, with either no AKI, hypovolaemia-induced AKI, or HRS-AKI, patients with HRS-AKI more often fulfilled criteria for systemic inflammatory response syndrome (SIRS) and had a significantly different cytokine profile compared to the other groups [15]. This was characterised by significantly higher levels of plasma IL-6, TNF-α, VCAM-1, and IL-8. The authors also found that the cytokine pattern of HRS-AKI was similar to that of other inflammatory conditions, including rheumatoid arthritis and inflammatory bowel disease. In paediatrics, there has been no direct characterisation of inflammation in HRS-AKI, but SIRS has been described as a risk factor for AKI of any aetiology in children with acute-on-chronic-liver failure [2].

Another important factor in the pathophysiology of HRS is the role of intra-abdominal pressure. The increasing volume of ascites leads to an increase in intra-abdominal pressure (IAP), which decreases renal blood flow through a direct increase in renal venous pressure [16]. This worsens renal perfusion, further leading to oliguria with rising renal markers.

A further contributor to HRS-AKI is cirrhotic cardiomyopathy, which refers to the structural and functional changes in the heart leading to either impaired systolic, diastolic, electrocardiographic, and neurohormonal changes associated with cirrhosis and portal hypertension. In other words, the heart does not pump blood as well as it should in patients with advanced cirrhosis. Cirrhotic cardiomyopathy is present in 26–81% of patients with cirrhosis [17], and leads to impaired contractility, diastolic dysfunction, a hyperdynamic circulation, and QT prolongation [18]. It has also been described in several paediatric studies [19, 20]. The end result of reduced cardiac reserve is a reduced mean arterial and renal perfusion pressure. These changes seem particularly marked following the development of infections and other acute events that lead to HRS-AKI.

Some children with biliary pathology also develop HRS-AKI. Cholemic nephropathy has long been reported in patients with cirrhosis and high serum bilirubin concentrations, and is thought to be caused by the formation of obstructing intratubular bile acid casts and direct bile acid toxicity to tubular cells [21]. Histopathologic studies have shown the presence of intratubular bile acid casts frequently in patients with HRS (in 11 out of 13 in one study [22]). High levels of urinary bilirubin and urobilinogen may be clues to the diagnosis. It is possible that cholemic nephropathy may be present in many patients with jaundice and HRS-AKI, and that it may affect outcomes and treatment response, but its role has been under-studied to date. Serum bilirubin concentrations above 10 mg/dL have been associated with an inferior response to vasoconstrictors in patients with HRS-AKI, compared with patients with serum bilirubin below 10 mg/dL (13% versus 67%; *p* = 0.001) [23]. Treatments targeting bile acids in patients with jaundice and HRS-AKI may therefore be beneficial. A recent study in an experimental model of biliary cirrhosis showed that administration of nor-ursodeoxycholic acid ameliorates renal function and histologic findings and may represent a therapeutic option for cholemic nephropathy [24]. Nevertheless, the pathophysiologic contribution of cholemic nephropathy to HRS-AKI has not been convincingly demonstrated and needs further investigation. Animal studies have shown that treatment with N-acetylcysteine (NAC) improves renal function in acute cholestasis [25]. Animal studies have also shown a potential mechanism by which NAC improves cardiac function [26]. Open studies of NAC in patients with HRS-AKI have also shown benefit in small numbers of patients [27], but no controlled studies have been undertaken.

## Hepatorenal syndrome in children

The most common indication for liver transplantation in children is biliary atresia. Biliary atresia is an inflammatory obliterative cholangiopathy which presents in early infancy with obstructive jaundice. The estimated prevalence of biliary atresia is between 1 in 15,000 and 19,000 livebirths [28]. The Kasai portoenterostomy is performed in infancy to re-establish bile flow, but despite this, most children with biliary atresia will require a liver transplantation before adulthood, with survival into adulthood with native liver varying between 14 and 44% [29]. Other common indications for liver transplantation in childhood include metabolic and genetic causes, acute liver failure, autoimmune liver diseases, and tumours [30]. As chronic liver disease progresses in these children, complications including portal hypertension, pruritus, vitamin deficiencies, and poor growth are common. As complications increase, in particular ascites and portal hypertension, so too does predisposition to HRS.

Unfortunately, specific paediatric data are lacking regarding the prevalence and outcomes of HRS-AKI. As mentioned, it is estimated that HRS-AKI occurs in around 5% of children with chronic liver disease prior to liver transplantation [3]. One study reported the prevalence of HRS-AKI to be 4.4% (16/367) amongst all children awaiting liver transplant, but there was an over-representation of these children amongst all children with dialysis-dependent AKI, with 16 of 30 (53%) [31]. A separate single-centre study of 84 children with ACLF found that 19 children (22.6%) developed AKI, of which six (31.6% of all cases with AKI) were diagnosed with HRS [2]. All six were managed with terlipressin and albumin. Four of these six children had poor outcomes, with two surviving after medical therapy alone, one requiring a liver transplant, and three dying.

Another key study of outcomes specifically focused on paediatric HRS was a paediatric case–control study of 8 children with HRS, who all were treated with kidney replacement therapy (KRT) for HRS while awaiting liver transplant, who were matched with 24 similar controls who required liver transplant but did not have HRS. In this study, the mortality rate for cases requiring KRT was 3/8 (37.5%), compared to 0% for controls without HRS [32]. All eight children with HRS experienced fluid overload with oligo/anuria, and 4/8 had electrolyte abnormalities, leading towards commencement of KRT. Children with HRS also more commonly had other complications associated with chronic liver disease, with 8/8 having ascites, 4/8 developing spontaneous bacterial peritonitis, 8/8 having gastrointestinal bleeding and 7/8 infections, all of which were significantly more common than in controls. Cases’ Pediatric End-Stage Liver Disease (PELD) or Model for End-Stage Liver Disease (MELD) scores, creatinine and bilirubin levels were also significantly elevated compared to controls. This study demonstrates that children with HRS are at significant risk of complications and poor outcomes.

Ultimately, the definitive treatment for children with HRS-AKI is a liver transplant. Adult studies suggest that liver transplantation effectively reverses HRS in around 80% of patients [33, 34]. However, liver transplantation confers its own risks and is not appropriate for all patients, so transplant candidates must be carefully evaluated [35, 36]. Encouragingly, limited paediatric data also suggest that there is the potential for renal recovery for children with HRS-AKI after liver transplant. The aforementioned case–control study reported that the median glomerular filtration rate for surviving cases post-transplant was not significantly different from that of controls at last follow-up, with a median GFR of 97 mL/minute/1.73 m^2^ versus 114 mL/minute/1.73 m^2^ in controls [32]. A small case series of four children with HRS from 1987 described that three out of four recovered to near-normal renal function post-transplant, with one patient having ongoing poor renal function [37].

## Hepatorenal syndrome in adults

In adults, the most common indications for liver transplantation are alcohol-associated liver disease, MASLD, and viral hepatitis [38]. HRS-AKI is better studied amongst the larger adult cohort. In a study of 2,063 adult patients admitted with AKI and cirrhosis across 11 US hospital networks in 2019, 12.1% (249/2063) had HRS-AKI, of which 242 (97%) had ascites, and 90-day mortality was high at 49% (122/249) [39]. In another study in adult patients with cirrhosis, patients with HRS-AKI had an odds ratio for 3-month mortality of 6.9 (95% confidence interval (CI) 2.2–21.6, *p* = 0.001) compared to other causes of renal failure [40]. These data underline the significance of HRS for an already sick cohort, whether adult or child.

## Terlipressin in the management of hepatorenal syndrome

As described, the definitive management for HRS-AKI is a liver transplant. In some high-risk cases, including those with HRS-AKI and acute tubular necrosis, simultaneous liver-kidney transplant may be considered [41]. However, organ transplants bring their own associated burdens, and not all patients are fit for transplant either due to severe critical illness or other contraindications [36]. The mainstay of medical management is terlipressin plus albumin, in addition to the usual reno-protective measures which we use in all critically unwell children with AKI (judicious use of fluids and caution with nephrotoxic drugs).

Terlipressin (triglycyl lysine vasopressin) is a synthetic analogue of vasopressin. It is metabolised to lysine vasopressin after N-triglycyl residue is cleaved by endothelial peptidases, leading to a prolonged release of lysine vasopressin, with an effect half-life of 6 h and prolonged reduction of portal venous pressures in pharmacological studies [42]. It has a particular affinity for the splanchnic circulation, causing splanchnic vasoconstriction and consequent reduction in portal pressures. It has been shown to be effective in adult trials for multiple indications including treatment of acute variceal bleeding [43], septic shock [44], and HRS-AKI. As previously described, in cirrhotic patients with HRS-AKI, as part of the pathological disease process, patients experience profound splanchnic vasodilatation, low systemic vascular resistance and mean arterial pressures, and activation of vasoconstrictor systems (including RAAS and the sympathetic nervous systems) leading to renal vasoconstriction and reduced GFR. In these patients, the effect of terlipressin, by inducing splanchnic vasoconstriction, leads to reduced splanchnic blood pooling, raised systemic vascular resistance and improved mean arterial pressures, reducing activation of the RAAS and sympathetic nervous system, and consequently reversal of renal vasoconstriction and improved renal perfusion pressure and blood flow [45]. Terlipressin therefore ultimately improves renal function in HRS-AKI.

The latest Cochrane Review on the use of terlipressin ± albumin in patients with cirrhosis and HRS was published in 2017. Across nine randomised controlled trials (RCTs) amongst participants mostly aged in their 50 s or 60 s, terlipressin had a beneficial effect on mortality (RR 0.85, 95% CI 0.73–0.98; 534 participants) and on reversal of hepatorenal syndrome (RR 0.63, 95% CI 0.48–0.82; 510 participants) compared to placebo or no intervention [46]. A 2018 network meta-analysis compared seven different therapeutic strategies for HRS and found terlipressin plus albumin showed a comprehensive effect, effective both at decreasing serum creatinine and increasing serum sodium [47].

In terms of route of administration, one RCT included in the review compared continuous intravenous infusion at an initial dose of 2 mg/day or intravenous boluses of terlipressin at an initial dose of 0.5 mg every 4 h [48]. There was no significant difference in the rate of response to treatment between groups, but the rate of adverse effects was significantly lower in the continuous infusion group (35% versus 62%, *p* < 0.025). The maximal daily terlipressin dose (2.62 ± 1.06 versus 4.5 ± 3.06 mg) and the mean daily dose (2.23 ± 0.65 versus 3.51 ± 1.77 mg) were also lower in the continuous infusion group, representing approximately 40% reduction in drug exposure. At our centre, we prefer to use a continuous infusion of terlipressin.

An additional key randomised, double-blind, placebo-controlled trial was published in 2021 – the CONFIRM trial [49]. This trial randomised 600 patients with HRS-1, cirrhosis, ascites, and rapidly progressive kidney failure across 60 sites in the United States and Canada in a 2:1 ratio to receive terlipressin or placebo for up to 14 days. Reversal of HRS occurred for 63 patients (32%) in the terlipressin group and 17 patients (17%) in the placebo group (*p* = 0.006). The analysis found that patients randomised to terlipressin also had a significantly shorter median length of stay in ICU compared to placebo (4 versus 11 days; *p* < 0.001) [50]. However, overall and transplantation-free survival at 90 days did not differ significantly between groups.

Subsequent to these data, as of 2024, terlipressin is now recommended as a first-line agent in the treatment of HRS-AKI by the ADQI and ICA [4]. The authors recommend commencing terlipressin in combination with 20–25% albumin immediately upon establishing a diagnosis of HRS-AKI, alongside close monitoring of volume status, with albumin to be discontinued if there is evidence of volume overload. Table 1 summarises the key aspects of using terlipressin in adults with HRS-AKI, extrapolated from the ADQI and ICA guidelines [4].

**Table 1:** Recommendations for use of terlipressin in adult HRS-AKI, extrapolated from 2023 ADQI and ICA guidelines [4]

	Recommendation
Initiation	Commence a vasoconstrictor (first line—terlipressin) immediately after diagnosis of HRS-AKI, in combination with 20–25% albumin
Dosing	Dose terlipressin as continuous infusion 2–12 mg/day or intravenous bolus 1–2 mg every 6 h Increase the dose of terlipressin every 24 h if serum creatinine has not decreased by 25% from baseline. Increase by at least 2 mg/day every 24 h for those on continuous infusion up to a maximum of 12 mg/day and increase from 1 to 2 mg every 6 h for IV bolus, if serum creatinine has not improved by 25%
Albumin	Assess fluid status. Unless overloaded, daily 20–25% albumin is recommended. Titrate based on volume status daily and discontinue if evidence of fluid overload or pulmonary oedema
Discontinuation	Discontinue for any of the following reasons: Serum creatinine within 0.3 mg/dL of baseline No improvement in serum creatinine after 48–72 h with maximal tolerated doses Serious adverse reaction Initiation of kidney replacement therapy Liver transplantation Total duration of 14 days

## Adverse effects and monitoring during treatment with terlipressin

Although there is consensus about the efficacy of terlipressin in the management of HRS-AKI, certain patients are more at risk of adverse effects, justifying caution and careful monitoring when prescribing. The 2017 Cochrane Review found that terlipressin did not increase the risk of serious adverse events as a composite outcome (RR 0.91, 95% CI 0.68–1.21; 534 participants) [46]. However, there was an increased risk of serious cardiovascular adverse events (RR 7.26, 95% CI 1.70–31.05). Other non-serious adverse effects analysed were diarrhoea and abdominal pain. Adverse effects, when they occur, tend to lead to treatment discontinuation early (within the first 7 days) [51].

One key adverse effect is the potential risk of respiratory failure, as observed in the CONFIRM trial, thought to be driven by fluid overload and pulmonary oedema. The 2021 CONFIRM trial identified that, although there was no overall difference in mortality, and no overall difference between groups in the proportion with serious adverse events (65% in the terlipressin group versus 61% in the placebo group), a higher percentage of patients in the terlipressin group experienced respiratory failure (10% versus 3%) [49]. Death by 90 days due to respiratory disorders occurred in 22 patients (11%) in the terlipressin group and 2 patients (2%) in the placebo group. The CONFIRM authors theorised that the trend towards negative respiratory outcomes may be due to direct cardiovascular and pulmonary effects of terlipressin. This effect has been previously reported; a 2010 study treated 24 patients with cirrhosis and ascites with terlipressin, and sought to evaluate the effects of terlipressin on cardiac function and perfusion [52]. The authors found that terlipressin led to a reduced cardiac output and ejection fraction but preserved myocardial perfusion. A post-hoc analysis by the CONFIRM study team in 2022 found that certain groups were more at risk of adverse effects, with 9.4% of patients with grade 1–2 ACLF developing respiratory failure compared to 30% with grade 3 ACLF (*p* = 0.0002) [53]. Additionally, in patients with grade 3 ACLF treated with terlipressin, multiple organ dysfunction syndrome and sepsis were slightly more common. Detailed narrative reviews have concluded that the trend in respiratory outcomes was driven by increased pulmonary oedema in a subgroup of patients who were at higher risk for this complication [54].

As a result of the above recent evidence, international regulatory agencies, including the UK Medicines and Healthcare products Regulatory Agency (MHRA), have issued specific recommendations to reduce risks of respiratory failure and septic shock in patients with HRS-AKI [55]. The MHRA advise avoiding the use of terlipressin in patients with severe renal dysfunction (baseline serum creatinine at or above 442 µmol/L) or severe liver disease (including ACLF grade 3) unless benefits outweigh risks.

Consequently, clinicians using terlipressin should aim to minimise complications through careful patient selection, judicious use of albumin after assessing the fluid status of each patient (avoiding albumin in euvolemic and hypervolemic patients), and regular monitoring for adverse effects. In practice in adults, the key is to ensure that patients are volume replete rather than overloaded, before starting terlipressin, and to commence terlipressin at lower doses first. Thus, increasingly, and especially since the Cavallin et al*.* RCT comparing continuous versus bolus terlipressin [48], centres use continuous infusions rather than bolus injection [56], and some advocate starting at 0.5 mg terlipressin QDS or 2 mg IV/24 h, rather than 1 mg four times daily or 4 mg/24 h in adults. This equates to considering a starting dose of 20 µg/kg/24 h in children, which equates to half the low dose given in adults, but can be increased stepwise depending on response, defined by changes in serum creatinine and monitoring for adverse effects.

## Terlipressin in paediatric HRS-AKI

There are few studies, and no RCTs, describing detailed outcomes following use of terlipressin for the treatment of HRS-AKI in children. It is better studied in the setting of refractory septic shock. A 2017 systematic review evaluating RCTs and clinical trials of vasopressors in neonates and children identified four studies describing use of terlipressin in cohorts of septic children, three via bolus dosing and one via continuous infusion [57]. All four described that terlipressin led to a significant increase in mean arterial pressures.

Amongst limited paediatric studies is a 2010 case series of four children with end-stage cirrhosis, ascites, and HRS who were treated with a continuous infusion of terlipressin (glypressin 30 μg/kg/day) and albumin (1 g/kg/day) [58]. Three of four had presentations compatible with HRS-AKI, and one with a nonacute form of HRS. The four patients also had improvement or stabilisation of renal function; one patient was bridged to liver transplant with normal renal function at last follow-up, but the other three died. No significant adverse effects were reported.

A study published in 2020 on the use of terlipressin in children with liver disease and AKI was published from 16 children treated in PICU at King’s College Hospital between 2010 and 2017 [59]. Seven of these 16 patients met criteria for HRS-AKI, although it was challenging to accurately discern HRS-AKI from non-HRS-AKI given the retrospective nature of this cohort and the ambiguity in the definition of HRS. Serum creatinine and urine output increased over time up to day 7 of terlipressin use, particularly amongst the subgroup with HRS-AKI. There was also an increase in mean arterial pressure. Three adverse effects occurred (two instances of digital ischaemia, one instance of feeding intolerance).

With regard to safety in children, data are also limited. In the aforementioned systematic review including four studies of terlipressin in septic shock [57], the most commonly reported adverse effects were tissue ischaemia/skin lesions and rhabdomyolysis, although how much these effects were sequelae of sepsis is unclear. In published guidance documents, contraindications are extrapolated from adult studies. Hyponatraemia has sometimes been reported to occur with terlipressin usage in other cohorts [60], but occurred in neither paediatric HRS study above. Regular serum sodium monitoring is recommended during treatment.

## Practical use of terlipressin in paediatric hepatorenal syndrome

**Fig. 1:**
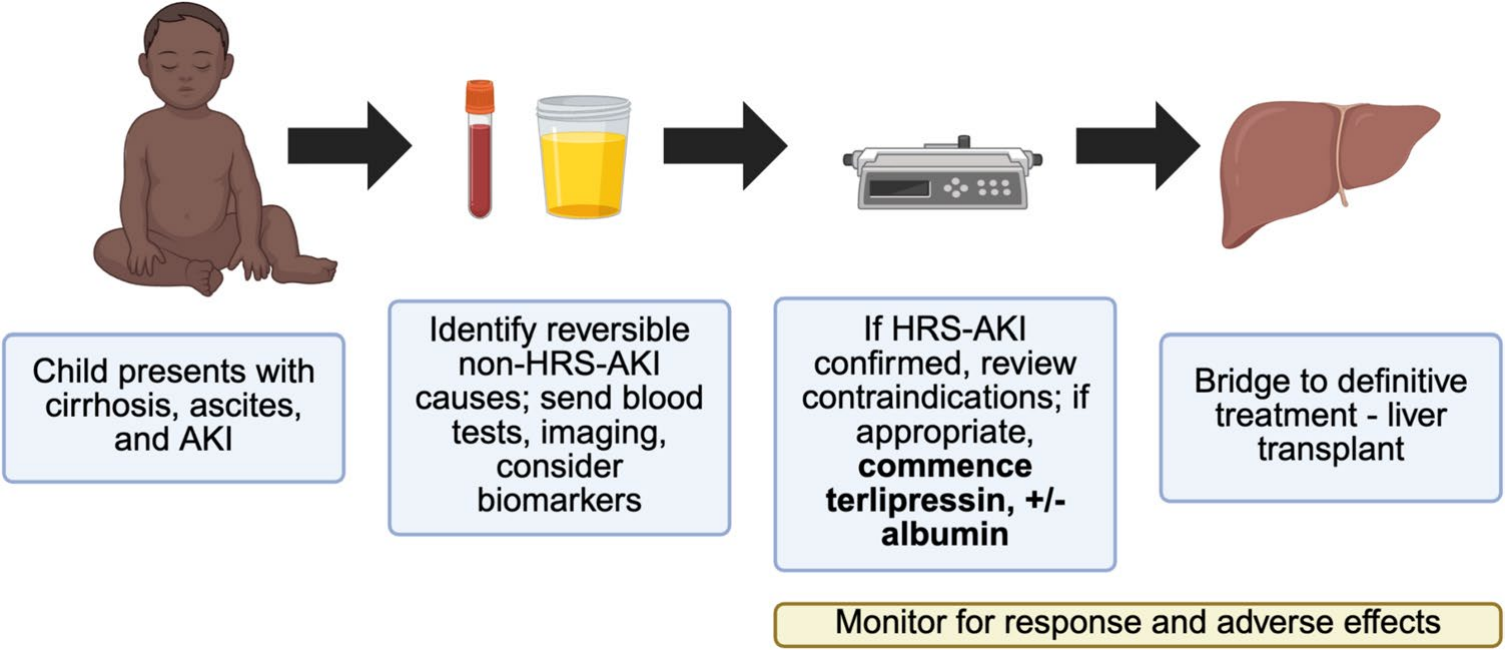
Flow chart for presentation of child and investigation and treatment for HRS-AKI. *Created in BioRender*

Based on the available literature and international clinical guidelines, we suggest general principles for the use of terlipressin, including monitoring recommendations, in children with HRS-AKI in Table 2. A flow chart illustrating potential assessment and management is shown in Fig. 1. The 14-day maximal duration is extrapolated from trials such as the CONFIRM trial which applied a planned treatment period of up to 14 days [49], rather than due to concerns of abrupt toxicity after this period. Complications from extended exposure are not known, though prolonged treatment can increase cumulative exposure to vasoconstrictive side effects. Paediatric data are limited, so duration should be individualised with frequent reassessment for response and adverse effects.

**Table 2:** Example protocol for use of terlipressin in children with HRS-AKI

**General principles**	Ensure the patient meets diagnostic criteria for HRS-AKI as defined by the 2023 Acute Disease Quality Initiative (ADQI) and International Club of Ascites (ICA) joint multidisciplinary consensus meeting [4] Treat any precipitating insult; stop nephrotoxic drugs and beta-blockers prior to commencing treatment
Cautions and contraindications	Severe HRS-AKI (Cr ≥ 442 µmol/L); ACLF grade 3; existing pulmonary oedema, respiratory failure or fluid overload; septic shock with low cardiac output; ischaemic heart disease; prolonged QT interval
Dosing	Initial dose 20 µg/kg/day (max 2 mg/24 h) Titrate to effect if required in increments of 5–10 µg/kg/day every 24 h following review Maximum of 60 µg/kg/day (max 5 mg/24 h)
Route	Preferably via a central line to reduce risk of extravasation injury
Albumin	Co-prescription with albumin of 0.5 g/kg per day is recommended Consider increasing dose of albumin if persistent hypovolaemia to 1 g/kg but ensure careful monitoring for signs of respiratory failure and pulmonary oedema Stop if any suspicion of pulmonary oedema
Dose adjustments	Dose should be increased in a stepwise manner as above if urine output does not increase to 30 ml/hr or > 1 ml/kg/hr or by > 100%, or serum creatinine does not decrease by at least 25% within 24 h, or if there is no pressor response as determined by an increase in mean arterial pressure of 15–20%/10 mmHg above the pre-initiation level after 24 h
Monitoring	Clinical vigilance for ischaemia, infection, and respiratory compromise is essential. Suggested monitoring on terlipressin includes: Daily labs: electrolytes (including sodium), renal function Lactate: every 4–6 hourly by blood gases Continuous vital signs and pulse oximetry, regular blood pressure ECG at baseline and in the event of new symptoms Monitor urine output Gastric aspirates for intolerance of feeds and abdominal examination for distension and absence of bowel sounds Frequent clinical assessment for ischaemia (looking at toes and fingertips), infection, and signs of respiratory distress/pulmonary oedema
Duration	Maximum duration of treatment is 14 days if treatment effective Discontinue if lack of response after 48–72 h on maximal doses Monitoring of response to terlipressin during continuous kidney replacement therapy may not be possible due to the effect of KRT on serum creatinine; consider discontinuing

Always refer to Product License for full list of cautions and contraindications

## Future research recommendations

It is clear, overall, that more research into HRS-AKI in children is needed. Most clinical guidelines extrapolate from adult data; however, children may not necessarily respond the same; the aetiology for chronic liver disease in children is very different. Risk factors for adverse effects while being treated with terlipressin, including pulmonary oedema, may be less prevalent in children, whereas the risks from extravasation, for example, may be comparatively higher in small children with difficult vascular access.

Given that management of children with cirrhosis is concentrated in a small number of centres, we recommend international collaborations to collate and quantify the outcomes of these patients. It will be important to determine both short- and long-term outcomes, including the risk of CKD both pre- and post-transplant. Adult studies suggest that terlipressin may lead to temporary improvement in renal function in patients with HRS-CKD; however, recurrence after treatment cessation is also common [61].

Future research should also incorporate evaluation of urinary biomarkers in any studies of paediatric HRS-AKI. In a study of 162 adults with cirrhosis and AKI, of which 40% had HRS-AKI, urinary neutrophil gelatinase-associated lipocalin (uNGAL) was shown to predict response to treatment with terlipressin and albumin amongst patients with HRS-AKI [62]. Therefore, if shown to be equally effective in paediatric patients, uNGAL could be used as one part of the risk-stratification process prior to starting children with HRS-AKI on terlipressin. Another promising biomarker is serum cystatin C, which has been characterised in a study of 62 children who underwent liver transplant, and shown to have high sensitivity and specificity as a biomarker of renal dysfunction, although it has not specifically been studied in HRS-AKI [63]. However, for both biomarkers, more research in practice is needed.

## Conclusions

Children with HRS-AKI are at high risk of morbidity and mortality. Liver transplantation is the mainstay of treatment, but medical management with terlipressin can bridge these patients to transplant, and some limited long-term data suggest long-term renal recovery post-transplant is possible. Careful selection of patients to treat is mandatory, starting with a low escalating dose of terlipressin with monitoring for adverse effects. International collaborations and efforts to publish data in this cohort, supplemented by further research into the predictive role of biomarkers, will improve outcomes for this high-risk group.

## Key summary points

1. Children with chronic liver disease are at high risk of acute kidney injury (AKI)
2. Identification of children with liver disease who develop hepatorenal syndrome-AKI (HRS-AKI) is of vital importance, given that data from both adults and paediatrics confirms these children are at increased risk of mortality and complications, and can also benefit from specific management strategies.
3. Other than liver transplantation, the key aspect of management is treatment with terlipressin, which has been shown to be effective in the treatment of HRS-AKI in randomised controlled trials conducted in adults, although in certain groups there are contraindications, including those with severe liver and renal failure and those at risk of pulmonary oedema.
4. Limited paediatric data validate a role for terlipressin in the management of paediatric HRS-AKI, but more research is urgently needed.

## Multiple choice questions

Answers are given following the references.

1. What is the primary pathophysiological mechanism underlying HRS-AKI?
  a. Glomerular inflammation
  b. Tubular obstruction by casts
  c. Renal vasoconstriction due to splanchnic vasodilation and systemic hemodynamic changes
  d. Direct renal damage from toxins
2. Which of the following criteria form part of the diagnosis of HRS-AKI?
  a. Presence of chronic kidney disease
  b. Evidence of intrinsic kidney injury on biopsy
  c. Lack of improvement in serum creatinine after adequate volume expansion (when clinically indicated)
  d. Elevated urine output despite increasing serum creatinine
3. Which of the following is the first-line pharmacological treatment in a child with hepatorenal syndrome (the child does not have severe liver or renal failure, and is not deemed at risk of pulmonary oedema)?
  a. Furosemide infusion
  b. Terlipressin and albumin
  c. Noradrenaline
  d. Haemodialysis

## Supplementary Information

Below is the link to the electronic supplementary material.

ESM 1 Graphical abstract (PPTX 190 KB)

## Data Availability

Data sharing is not applicable to this article as no datasets were generated or analysed during the current paper.
